# H_2_-Annealed Core–Shell Pt-Co/C Electrocatalysts with Enhanced Durability for the Oxygen Reduction Reaction

**DOI:** 10.3390/ma19214689

**Published:** 2026-11-04

**Authors:** Bingguo Xue, Wei Wang, Yuxiang Dai, Shaohong Liu, Limin Zhou, Hao Cui, Hongying Pei, Wenyan Zhou, Manmen Liu, Haigang Dong, Ming Wen, Li Chen, Song Li

**Affiliations:** 1 Key Laboratory for Anisotropy and Texture of Materials (Ministry of Education), School of Materials Science and Engineering, Northeastern University, Shenyang 110819, China; 2 State Key Laboratory of Precious Metal Functional Materials, Yunnan Precious Metals Laboratory Co., Ltd., Kunming 650106, China; 3 Sino-Platinum Metals Semiconductor Materials (Yunnan) Co., Ltd., Kunming 650106, China

**Keywords:** oxygen reduction reaction, PtCo alloy, hydrogen annealing, Pt-rich shell, hierarchically porous carbon, proton exchange membrane fuel cell

## Abstract

**Highlights:**

- Hydrogen annealing reverses Co segregation to build a Pt-rich shell on PtCo/C.
- Mesopore confinement blocks particle coalescence during accelerated testing.
- Pt_3_Co_1_/C matches Pt/C ORR activity with one quarter of metal atoms Co.
- Half-wave potential decays only 48 mV after 10,000 durability cycles.
- Pt-rich shell suppresses Co leaching without hollowing or lattice relaxation.

**Abstract:**

The oxygen reduction reaction (ORR) at the cathode remains a major kinetic bottleneck for proton-exchange-membrane fuel cells, and Pt-Co alloying is a well-established strategy for boosting Pt-mass-normalized ORR activity. However, the long-term durability of such alloys is limited by two largely independent degradation pathways, dealloying-induced Co leaching and nanoparticle coalescence, that are rarely addressed simultaneously. Here, a series of Pt_x_Co_y_/C-H_2_ catalysts with varying Pt:Co ratios were synthesized via gel-confined co-reduction of Pt and Co precursors, phosphoric-acid-assisted carbonization into a mesoporous carbon support, and a subsequent H_2_-annealing step. XRD confirmed Pt-Co alloy formation with composition-dependent lattice contraction, while XPS showed that surface Pt:Co ratios consistently exceeded the nominal feed ratios across all compositions, indicating H_2_-annealing-induced Pt surface segregation and the formation of a Pt-enriched, core–shell-like architecture, further supported by TEM/EDS line-scan profiles. Among the series, Pt_3_Co_1_/C-H_2_ delivered ORR activity (E_1/2_, mass activity, and specific activity) essentially comparable to commercial Pt/C while reducing Pt loading by approximately 9 wt%, and retained the intrinsic four-electron ORR pathway (*n* = 3.78). After accelerated durability testing (10,000 cycles), Pt_3_Co_1_/C-H_2_ exhibited smaller E_1/2_ decay than Pt/C (48 vs. 52 mV), which is attributed to the concurrent suppression of nanoparticle coalescence by mesopore confinement and of Co leaching by the Pt-enriched surface. These results establish a generalizable design principle for decoupling the dominant degradation pathways of Pt-transition-metal alloy ORR catalysts while reducing precious-metal content.

## 1. Introduction

Proton-exchange-membrane fuel cells (PEMFCs) are regarded as one of the most promising clean-energy conversion technologies, owing to their high energy-conversion efficiency, zero on-site emissions, and rapid start-up characteristics [1,2,3]. However, the sluggish kinetics of the oxygen reduction reaction (ORR) at the cathode remain a major bottleneck limiting the large-scale commercialization of PEMFCs [4,5,6]. Pt-based materials are currently the only catalysts that combine sufficient ORR activity with the stability required under the strongly acidic [7,8,9,10,11], oxidizing operating environment, but the scarcity and high cost of Pt account for a substantial fraction of total stack cost, making the reduction of Pt loading without sacrificing activity or durability a central challenge in PEMFC catalyst development [12,13,14].

Alloying Pt with 3d transition metals (Fe, Co, Ni, etc.) has proven to be an effective strategy for addressing this challenge [15,16,17,18]. Incorporation of a smaller transition-metal atom into the Pt lattice induces lattice contraction and downshifts the Pt d-band center, which weakens the binding of oxygenated intermediates and thereby enhances the intrinsic ORR activity per unit Pt mass [19,20,21]. Among these systems, Pt-Co alloys have attracted particular attention because of their favorable balance between activity enhancement and synthetic accessibility, and Pt-Co/C catalysts with several-fold higher mass activity than commercial Pt/C have been reported [22].

Despite these activity gains, the long-term durability of Pt-transition-metal alloy catalysts under realistic operating conditions remains a critical concern, and is governed by two largely independent degradation pathways [23]. The first is dealloying: the non-noble transition metal, being thermodynamically less stable in acidic media, undergoes continuous leaching from the alloy surface, which not only depletes the alloying element responsible for the electronic modification but also roughens the particle surface and accelerates further corrosion [24]. The second is nanoparticle coalescence (via migration/agglomeration or Ostwald ripening), which reduces the electrochemically active surface area and is largely independent of the alloy’s chemical composition [25]. Because these two pathways originate from distinct physicochemical origins, chemical and structural, respectively, strategies that target only one of them tend to leave the catalyst vulnerable to the other [26].

Two design strategies have been widely explored to address these pathways individually [27]. Core–shell or Pt-skin architectures, in which a Pt-enriched outer layer covers a Pt-M alloy core, suppress dealloying by limiting the direct exposure of the transition metal to the electrolyte, but such structures alone do not prevent particle coalescence during long-term cycling [28]. Conversely, confining metal nanoparticles within a porous carbon matrix restricts their mobility and mitigates coalescence, but does not address compositional instability at the alloy surface [29]. A catalyst architecture that simultaneously incorporates both features, surface Pt enrichment and physical confinement within a porous support, would therefore be expected to decouple and suppress both degradation mechanisms concurrently, yet this combined strategy has been comparatively less explored [30].

In this work, we report a series of Pt_x_Co_y_/C-H_2_ catalysts, prepared via a gel-confined co-reduction of Pt and Co precursors within a sodium polyacrylate matrix, followed by phosphoric-acid-assisted carbonization to generate a mesoporous carbon support and a subsequent H_2_-annealing step to drive surface Pt segregation. This synthesis integrates mesopore-confined alloy nanoparticles with an H_2_-annealing-induced, Pt-enriched near-surface region within a single architecture. By systematically varying the Pt:Co feed ratio, we investigate the composition-dependent evolution of structure, particle architecture, and ORR activity and durability, and identify an optimal composition, Pt_3_Co_1_/C-H_2_, that matches the activity of commercial Pt/C at reduced Pt content while exhibiting improved durability through the concurrent suppression of coalescence and dealloying. This work provides both a practical Pt-lean cathode catalyst candidate and a generalizable design principle for decoupling the dominant degradation pathways in Pt-transition-metal alloy catalysts.

## 2. Materials and Methods

### 2.1. Catalyst Synthesis

All chemicals were of analytical grade and purchased from Sinopharm Chemical Reagent Co., Ltd. (Shanghai, China), and used as received. Ultrapure water (18.0 MΩ cm) was used throughout. H_2_PtCl_6_⋅6H_2_O and CoCl_2_⋅6H_2_O (total metal 1 mmol) were dissolved in 50 mL of deionized water at nominal Pt:Co atomic ratios of 3.5:1, 3:1, 2:1, 1.5:1, 1:1, and 1:3, after which 1 g of sodium polyacrylate resin was added under stirring to form a homogeneous gel. An aqueous NaBH_4_ solution (1 mol·L^−1^, 10 mL) was added dropwise at 25 °C to reduce the metal precursors in situ within the gel matrix, and the gel was freeze-dried for 24 h. The resulting powder was impregnated with 10 mL of 1 mol·L^−1^ phosphoric acid, oven-dried at 100 °C, carbonized at 800 °C for 2 h under flowing Ar (25 mL·min^−1^, ramp 10 °C·min^−1^), and subsequently calcined in static air at 300 °C for 3 h. After washing with deionized water to neutral pH, filtration, and drying, the intermediates were reduced at 300 °C for 1 h under flowing H_2_ (80 vol.% in Ar, 50 mL·min^−1^) to yield the Pt_x_Co_y_/C-H_2_ catalysts.

### 2.2. Physical Characterization

XRD patterns were recorded on a PANalytical X’Pert PRO diffractometer (PW 3040/60 goniometer, PANalytical B.V., Almelo, The Netherlands) with Cu Kα radiation (*λ* = 1.54056 Å) at 40 kV and 200 mA over 2*θ* = 10–90° (scan rate 2°·min^−1^). Morphology was examined by field-emission SEM (JSM-7001F, JEOL Ltd., Tokyo, Japan) equipped with EDS. Microstructure and elemental distribution were analyzed by TEM/HRTEM (Tecnai G2 F20, FEI Company, Hillsboro, OR, USA, 200 kV) with EDS line scans and elemental mapping; samples were ultrasonically dispersed in ethanol and drop-cast onto carbon-coated Cu grids. XPS was performed on a Thermo Escalab 250Xi spectrometer (Thermo Fisher Scientific, Waltham, MA, USA) (Al Kα, 1486.6 eV), with binding energies referenced to the C 1s peak at 284.8 eV. N_2_ physisorption isotherms were measured at 77 K on a Micromeritics ASAP 2020 analyzer (Micromeritics Instrument Corp., Norcross, GA, USA) after degassing at 200 °C for 2 h; specific surface areas were obtained by the BET method, mesopore size distributions by BJH analysis of the desorption branch, and micropore volumes by the HK method. Raman spectra were acquired on an inVia Raman spectrometer (Renishaw plc, Wotton-under-Edge, UK) (532 nm excitation, 5 mW, 10 s acquisition).

### 2.3. Electrochemical Measurements

Electrochemical measurements were performed on a CHI-760D/E workstation (CH Instruments, Inc., Shanghai, China) in a standard three-electrode rotating-disk-electrode (RDE) configuration at room temperature. A 3 mm glassy-carbon RDE (ALS Co., Ltd., Tokyo, Japan) served as the working electrode, a carbon rod as the counter electrode, and an Hg/Hg_2_SO_4_ electrode (filled with saturated K_2_SO_4_ solution) as the reference electrode. The electrolyte was 0.1 M HClO_4_. Potentials were converted to the reversible hydrogen electrode (RHE) scale according to *E*_RHE_ = *E*(Hg/Hg_2_SO_4_) + 0.0592 pH + 0.656 V. The disk was polished with 0.05 μm Al_2_O_3_. Catalyst ink was prepared by sonicating 5 mg of catalyst in 600 μL water and 200 μL ethanol for 1 h, adding 50 μL of 5 wt.% Nafion solution, and sonicating for a further 1 h. A measured aliquot was drop-cast on the disk and dried while rotating at 800 rpm. Commercial Pt/C (20 wt.% Pt, Tanaka Kikinzoku Kogyo K.K., Tokyo, Japan) was tested as the benchmark. Electrodes were activated in N_2_-saturated 0.1 M HClO_4_ by cycling from 0.05 to 1.20 V at 100 mV·s^−1^ until a stable voltammogram was obtained. CVs were then recorded from 0.05 to 1.10 V at 50 mV·s^−1^ without rotation. ECSA was calculated from the double-layer-corrected H_upd_ charge integrated over 0.08–0.40 V using 210 μC·cm^−2^ for a monolayer of adsorbed hydrogen and normalized to Pt loading. The double-layer capacitance (C_dl_) was determined from double-layer charging currents measured by cyclic voltammetry (CV) in N_2_-saturated 0.1 M HClO_4_ electrolyte. ORR LSVs were measured in O_2_-saturated 0.1 M HClO_4_ at 5 mV·s^−1^ and 1600 rpm. Rotation-dependent LSVs at 400, 900, 1225, 1600, 2025, and 2500 rpm were analyzed with the Koutecký–Levich equation to obtain the electron-transfer number n. Tafel plots and electrochemical impedance spectroscopy were used to assess reaction kinetics and charge transfer. EIS measurements were performed at 0.85 V vs. RHE, with the rotating disk electrode maintained at 1600 rpm throughout the measurements. The EISs were fitted using the equivalent circuit R_s_–(R_1_//C_1_)–(R_2_//C_2_)–W_1_, where R_s_ represents the solution resistance, R_1_ is associated with interfacial/contact processes within the catalyst layer, potentially including electronic transport, R_2_ represents the charge-transfer resistance of the ORR at the catalyst/electrolyte interface, C_1_ and C_2_ are the corresponding capacitances, and W_1_ is a Warburg element accounting for diffusion. Mass activity and specific activity were extracted at 0.85 V by normalizing kinetic current to Pt loading and ECSA, respectively. Accelerated degradation tests comprised 10,000 cycles between 0.60 and 1.00 V at 100 mV·s^−1^ in O_2_-saturated 0.1 M HClO_4_; the electrolyte was replaced every 5000 cycles, after which N_2_-saturated CVs and O_2_-saturated polarization curves were collected. Electron-transfer numbers were obtained from the rotation-dependent RDE data using Koutecký–Levich analysis.

## 3. Results

### 3.1. Morphology and Pore Structure

The porous carbon support, prepared by freeze-drying the gel-polymerized precursor followed by phosphoric acid impregnation and high-temperature carbonization, exhibits irregularly shaped particles ranging from hundreds of nanometers to several micrometers (Figure 1a–f). Metal nanoparticles formed via in situ reduction are uniformly dispersed throughout the carbon matrix without observable agglomeration (Figure 1a–f). Nitrogen physisorption analysis (Figure 2) reveals a comparable BET surface area of 540 m^2^·g^−1^ for all samples, confirming excellent structural stability with negligible collapse during thermal treatments. The BJH pore-size distribution demonstrates a mesopore-dominated structure, with pore diameters centered at ~10 nm accounting for over 90% of the total pore volume, while micropores contribute less than 5%. This mesoporous architecture, featuring individual pore dimensions comparable to the metal particle sizes, creates effective carbon-framework compartments that spatially confine neighboring particles and suppress long-range sintering—a structural characteristic crucial for the enhanced durability.

The Raman spectra of all six catalysts (Figure 3) exhibit two prominent bands: the D band at 1350 cm^−1^, associated with structural defects and disordered carbon domains, and the G band at 1590 cm^−1^, corresponding to the in-plane vibration of sp^2^-hybridized carbon. The intensity ratio *I_D_*/*I_G_*, which is inversely correlated with the degree of graphitization, remains nearly invariant across the Pt:Co composition series. This indicates that the carbon support retains a consistent defect density and microstructure regardless of the Pt:Co ratio, suggesting that variations in catalytic performance among the six samples originate primarily from differences in the metal-phase composition rather than from the carbon support itself.

### 3.2. Crystal Structure and Surface Composition

X-ray diffraction (XRD) patterns of the six Pt_x_Co_y_/C catalysts (Figure 4) reveal five characteristic diffraction peaks corresponding to the (111), (200), (220), (311), and (222) planes of the face-centered cubic (fcc) Pt-based structure. Depending on the Pt:Co ratio, these peaks appear within the 2θ ranges of 39.9–41.9°, 46.2–48.0°, 67.5–69.3°, 81.3–83.1°, and 85.8–87.5°, respectively. As the nominal Pt:Co ratio decreases from 3.5:1 to 1:3, the (111) peak shifts progressively to higher angles, from 40.1° to 41.8°, with the corresponding lattice parameter decreasing from approximately 3.89 to 3.74 Å (a contraction of approximately 4.6% relative to pure Pt, a = 3.92 Å), consistent with the formation of Pt-Co alloys in which the smaller Co atoms (r_Co_ = 1.25 Å) substitute into the Pt lattice (r_Pt_ = 1.39 Å), inducing lattice contraction. Notably, Pt_3_Co_1_/C, which delivers the best ORR performance among the series (Section 3.4), exhibits a (111) peak position closely matching that of the Pt_3_Co reference (PDF#01-072-9178), confirming the successful formation of the targeted alloy stoichiometry.

Transmission electron microscopy (TEM) images (Figure 5a) show that the metal nanoparticles are quasi-spherical, with a narrow size distribution and good dispersion on the carbon support. Energy-dispersive X-ray spectroscopy (EDS) line-scan profiles across individual particles (Figure 5b,c) reveal that the Pt and Co signals overlap spatially, with the Pt intensity slightly elevated at the particle periphery, consistent with the formation of a Pt-enriched shell. Based on the width of the Pt-enriched region in the line-scan profile, the shell thickness is estimated to be approximately 1 nm. High-resolution TEM (HRTEM, Figure 5d) resolves lattice fringes with d-spacings of 0.223 nm and 0.191 nm, corresponding to the (111) and (200) planes of the fcc alloy phase, respectively, with an interplanar angle of 54.7°, in good agreement with the cubic symmetry expected for this structure. This is further corroborated by the corresponding fast Fourier transform (FFT) pattern (Figure 5e), which displays diffraction spots consistent with the fcc structure viewed along the [011] zone axis. Elemental mapping images (Figure 5f–h) show overlapping Pt and Co distributions within the particles, confirming the alloyed nature of the nanoparticles. Taken together, the line-scan and mapping results support a core–shell-like structure with a Pt-enriched shell (~1 nm thick) surrounding a PtCo alloy core.

X-ray photoelectron spectroscopy (XPS) was employed to probe the surface chemical states and near-surface composition of the Pt_x_Co_y_/C-H_2_ catalysts. The survey spectra (Figure 6) confirm the presence of Pt, Co, C, and O in all samples. Since XPS probes only the top few nanometers of the particle surface, the derived elemental ratios reflect the near-surface composition rather than the bulk alloy stoichiometry. Notably, the XPS-derived surface Pt:Co ratios of all six catalysts (ranging from 2.60:1 to 6.76:1, Table S1) are consistently higher than the corresponding nominal feed ratios (0.33:1–3.5:1) and those of the Pt_x_Co_y_/C-Air intermediates before H_2_ annealing (ranging from 0.28:1 to 4.15:1, Table S2), providing direct evidence of Pt enrichment in the near-surface region. This surface Pt segregation is in good agreement with the TEM-EDS line-scan results (Figure 5b,c), which reveal elevated Pt signals at the particle periphery, and can be attributed to thermodynamically driven Pt surface segregation during H_2_ annealing, owing to the lower surface energy of Pt relative to Co. Taken together, the XPS and EDS results consistently point to a core–shell-like architecture with a Pt-enriched near-surface region surrounding a PtCo alloy core.

The high-resolution Pt 4f spectra (Figure 7) exhibit the characteristic spin–orbit doublet dominated by metallic Pt^0^, together with a minor Pt^2+^ component, with Pt^0^ accounting for 52.19–65.53% of the total Pt 4f intensity across the series, indicating that Pt remains predominantly metallic after H_2_ annealing. With increasing Co content, the Pt 4f_7/2_ binding energy shifts progressively from 71.73 eV (Pt_3.5_Co_1_/C-H_2_) to 71.50 eV (Pt_1_Co_3_/C-H_2_), with intermediate values of 71.69, 71.65, 71.60, and 71.56 eV for Pt_3_Co_1_/C-H_2_, Pt_2_Co_1_/C-H_2_, Pt_1.5_Co_1_/C-H_2_, and Pt_1_Co_1_/C-H_2_, respectively, showing a progressive negative shift of up to 0.23 eV and indicating enhanced electronic interaction between Pt and Co. Given the lower electronegativity of Co (1.88) relative to Pt (2.28), this negative shift is ascribed to partial electron transfer from Co to Pt, which increases the electron density of surface Pt atoms. Together with the lattice contraction revealed by XRD (Figure 4), such electronic and strain effects are expected to downshift the Pt d-band center and weaken the adsorption of oxygenated intermediates, thereby benefiting the ORR kinetics (Section 3.4).

The Co 2p spectra (Figure 8) display two main signals at approximately 780 and 796 eV, assigned to Co ^2^p_3/2_ and Co ^2^p_1/2_, respectively, accompanied by pronounced shake-up satellites characteristic of oxidized Co species. A weak Co^0^ component near 778 eV is also resolved, suggesting the presence of metallic Co within the alloy. The predominance of oxidized Co on the surface, despite the reductive H_2_ treatment, is attributed to the inevitable reoxidation of surface-exposed Co upon air exposure during sample transfer. Importantly, this surface oxidation does not compromise the alloy framework, as evidenced by the XRD and TEM results discussed above.

The C 1s spectra (Figure 9) are dominated by a peak at 284.8 eV assigned to graphitic (sp^2^) carbon, together with weaker components at higher binding energies attributable to oxygen-containing functional groups (C-O, C=O, and O-C=O). These oxygenated surface groups, together with the partially oxidized Co species, account for the O signal observed in the survey spectra. No obvious variation in carbon surface chemistry is observed across the composition series, indicating that the carbon support remains chemically comparable among the six catalysts, consistent with the Raman results (Figure 3).

### 3.3. Electrochemical Performance

The electrochemical behavior of the Pt_x_Co_y_/C-H_2_ catalysts was first characterized by cyclic voltammetry (CV) in N_2_-saturated 0.1 M HClO_4_ (Figure 10a). All samples display the characteristic voltammetric features of polycrystalline Pt: hydrogen underpotential deposition (H_upd_) peaks between 0.08 and 0.40 V, a double-layer region between 0.40 and 0.60 V, and the reversible formation and reduction of surface Pt oxides above 0.75 V, all referenced to RHE. After double-layer correction, the H_upd_ charge was integrated and converted to the electrochemically active surface area (ECSA) using a specific charge of 210 μC·cm_Pt_^−2^, yielding values of 126.9 m^2^·g_Pt_^−1^ for Pt_3_Co_1_/C-H_2_ and 130.3 m^2^·g_Pt_^−1^ for commercial Pt/C, indicating comparable Pt utilization between the two catalysts.

Linear sweep voltammetry (LSV) in O_2_-saturated 0.1 M HClO_4_ (Figure 10b) yields well-defined sigmoidal polarization curves, with diffusion-limited current densities ranging from approximately 4.2 to 5.3 mA·cm^−2^ across the catalyst series. The optimized Pt_3_Co_1_/C-H_2_ catalyst exhibits an onset potential of 0.936 V (at 0.1 mA·cm^−2^) and a half-wave potential (E_1/2_) of 0.842 V vs. RHE, essentially comparable to commercial Pt/C (0.937 V and 0.850 V) under identical conditions, with the 8 mV difference in E_1/2_ falling within the typical run-to-run variation in rotating-disk electrode measurements. In contrast, Pt_1_Co_1_/C-H_2_ shows a more pronounced decline (0.896 V and 0.827 V), consistent with a progressive loss of activity on the Co-rich side of the composition series. This trend suggests that, beyond an optimal Co fraction, the electronic benefit of Pt-Co alloying is outweighed by the reduction in the number of accessible Pt active sites.

The corresponding Tafel slopes (Figure 10c) are 109 mV·dec^−1^ for Pt_3_Co_1_/C-H_2_ and 97 mV·dec^−1^ for commercial Pt/C. Both values are reasonably close to the theoretical value of ~120 mV·dec^−1^ expected when the first electron transfer is rate-determining under Temkin-type surface oxide coverage, indicating that Co incorporation does not alter the intrinsic ORR mechanism [31].

Electrochemical impedance spectroscopy (EIS, Figure 10d) at 0.85 V vs. RHE gives a charge-transfer resistance (R_ct_) of 10.5 Ω for Pt_3_Co_1_/C-H_2_ versus 9 Ω for commercial Pt/C, consistent with the activity ranking obtained from LSV. The double-layer capacitance (*C*_dl_), extracted from the scan-rate dependence of the non-Faradaic current (Figure 10e), is 26.7 mF·cm^−2^ for commercial Pt/C and ranges from 23.8 to 34.7 mF·cm^−2^ across the Pt_x_Co_y_/C-H_2_ series (34.7 mF·cm^−2^ for Pt_3_Co_1_/C-H_2_). The near-invariant *C*_dl_ across the composition series confirms that the electrochemically accessible carbon surface area is preserved after H_2_ annealing, consistent with the BET and Raman results (Section 3.1).

### 3.4. Activity Metrics and the Effect of Reduced Pt Content

To quantify performance on an absolute scale, the kinetic current density at 0.85 V vs. RHE was normalized by Pt mass (mass activity, MA) and by ECSA (specific activity, SA). Pt_3_Co_1_/C-H_2_ delivers an MA of 0.10 A·mg_Pt_^−1^ and an SA of 0.27 mA·cm_Pt_^−2^, corresponding to 1.04 and 1.05 times the respective values of commercial Pt/C (Figure 10f). These results are consistent with the essentially comparable E_1/2_ values noted above (Section 3.3), confirming that the alloy catalyst matches the intrinsic activity of the Pt/C benchmark.

The significance of these figures lies in the Pt loading at which they are achieved. In Pt_3_Co_1_/C, one quarter of the metal atoms (25 at.%) are replaced by Co, corresponding to a composition of approximately 90.9 wt% Pt and 9.1 wt% Co, roughly 9 wt% less Pt per gram of metal than a pure-Pt catalyst. The outcome is therefore not exceptional activity, but parity with a state-of-the-art Pt/C benchmark achieved at a reduced noble-metal inventory. Since Pt loading is the primary cost driver for a PEMFC cathode, this equivalence at lower Pt content reflects the practical value of the alloying strategy.

The apparent electron-transfer number extracted from rotating disk electrode (RDE) measurements using Koutecký–Levich (K-L) analysis (Figure 11a,b) is *n* = 3.78 for Pt_3_Co_1_/C-H_2_, consistent with predominantly four-electron ORR behavior, comparable to that of commercial Pt/C.

### 3.5. Durability and Post-Mortem Analysis

Catalyst durability was assessed by an accelerated durability test (ADT) comprising 10,000 potential cycles between 0.60 and 1.00 V at 100 mV·s^−1^ in O_2_-saturated 0.1 M HClO_4_, with the electrolyte renewed every 5000 cycles (Figure 11c). After the ADT, Pt_3_Co_1_/C-H_2_ retains an E_1/2_ of 0.793 V, corresponding to a decay of 48 mV, less than the 52 mV loss observed for commercial Pt/C under identical conditions.

Post-mortem TEM (Figure 11d–f) reveals that the mean particle size increases from 10.17 to 17.28 nm over the course of the ADT, indicative of particle coarsening. Two structural features of the present catalyst architecture help mitigate further degradation. First, the mesopore-dominated carbon framework (Section 3.1) physically separates neighboring particles at a length scale comparable to the particle diameter, geometrically frustrating direct particle–particle contact and thereby suppressing the coalescence pathway. Coarsening is consequently restricted to the slower dissolution–redeposition (Ostwald ripening) channel. Second, the Pt-enriched near-surface region formed during H_2_ annealing (Section 3.2) presents a Co-depleted outermost surface to the electrolyte, suppressing the selective Co dissolution that typically drives the degradation of conventional PtCo alloy catalysts. Consistent with this, post-mortem TEM shows no evidence of hollow-particle formation, porous-shell development, or lattice relaxation, features that typically accompany extensive Co leaching from the particle interior, although morphological evidence alone cannot exclude limited near-surface Co loss.

## 4. Conclusions

In summary, a series of Pt_x_Co_y_/C-H_2_ electrocatalysts with varying Pt:Co ratios were synthesized and shown to form Pt-Co alloys with lattice contraction, while H_2_ annealing drove surface Pt segregation and produced a core–shell-like, Pt-enriched surface architecture. Among the series, Pt_3_Co_1_/C-H_2_ delivered ORR activity (E_1/2_, MA, and SA) essentially comparable to commercial Pt/C while reducing the Pt loading by about 9 wt%, and it retained the intrinsic four-electron ORR pathway of Pt/C, confirming that alloying with Co is an effective strategy for improving Pt utilization without sacrificing activity.

Accelerated durability testing further showed that Pt_3_Co_1_/C-H_2_ exhibited smaller E_1/2_ decay than Pt/C (48 vs. 52 mV) after 10,000 cycles, which we attribute to two decoupled and complementary mechanisms: the mesoporous carbon support geometrically confines the Pt-Co nanoparticles and suppresses their coalescence, while the Pt-enriched surface layer limits direct exposure of Co and mitigates dealloying-induced Co leaching. Together, these structural and compositional features decouple the two dominant degradation pathways of alloy catalysts, offering a practical design strategy for durable, Pt-lean cathode catalysts in PEMFCs and other Pt–transition-metal alloy systems.

## Figures and Tables

**Figure 1:**
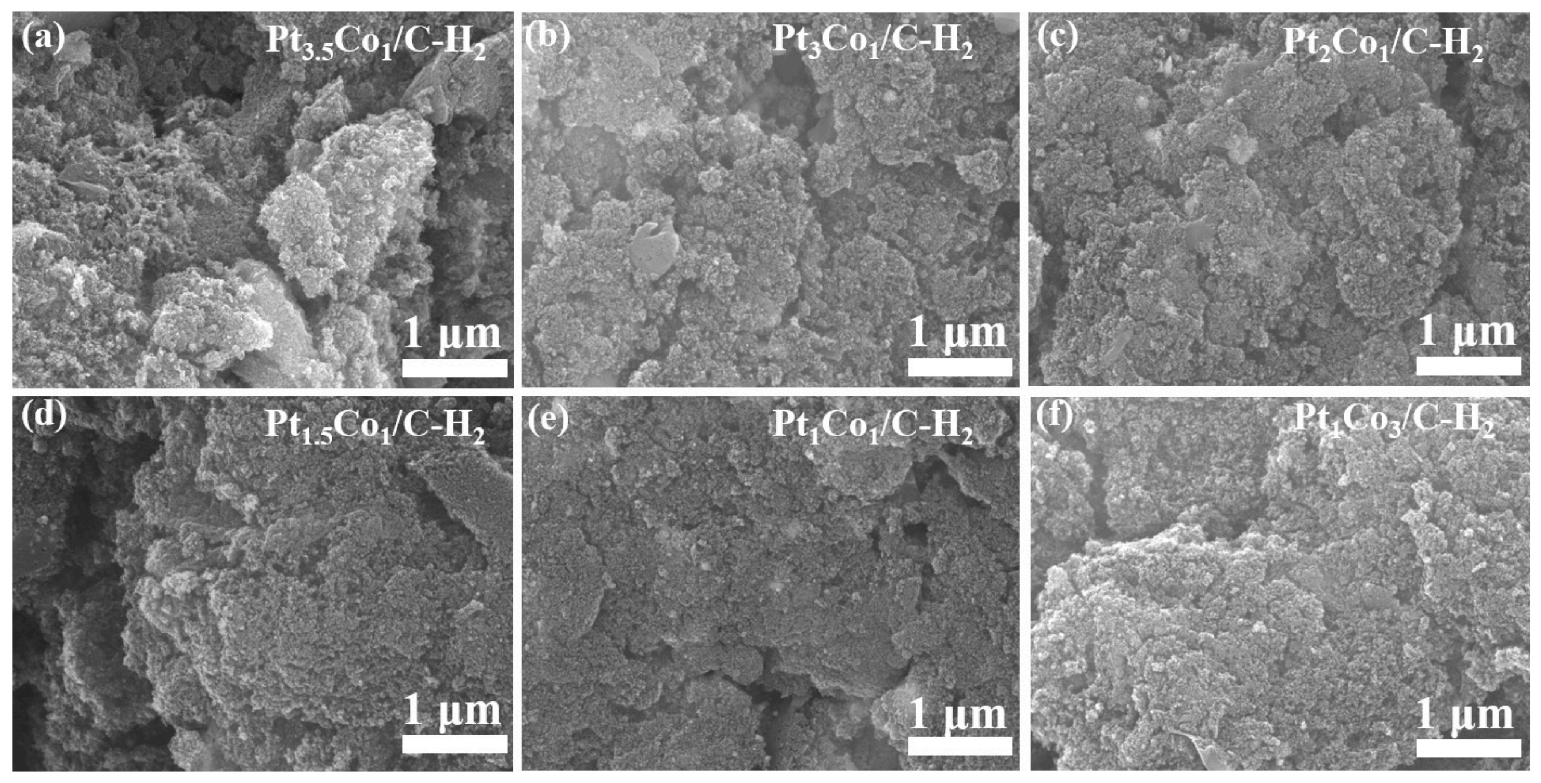
SEM images of Pt_x_Co_y_/C-H_2_ catalysts with varying Pt:Co ratios: (**a**) Pt_3.5_Co_1_/C-H_2_, (**b**) Pt_3_Co_1_/C-H_2_, (**c**) Pt_2_Co_1_/C-H_2_, (**d**) Pt_1.5_Co_1_/C-H_2_, (**e**) Pt_1_Co_1_/C-H_2_, and (**f**) Pt_1_Co_3_/C-H_2_.

**Figure 2:**
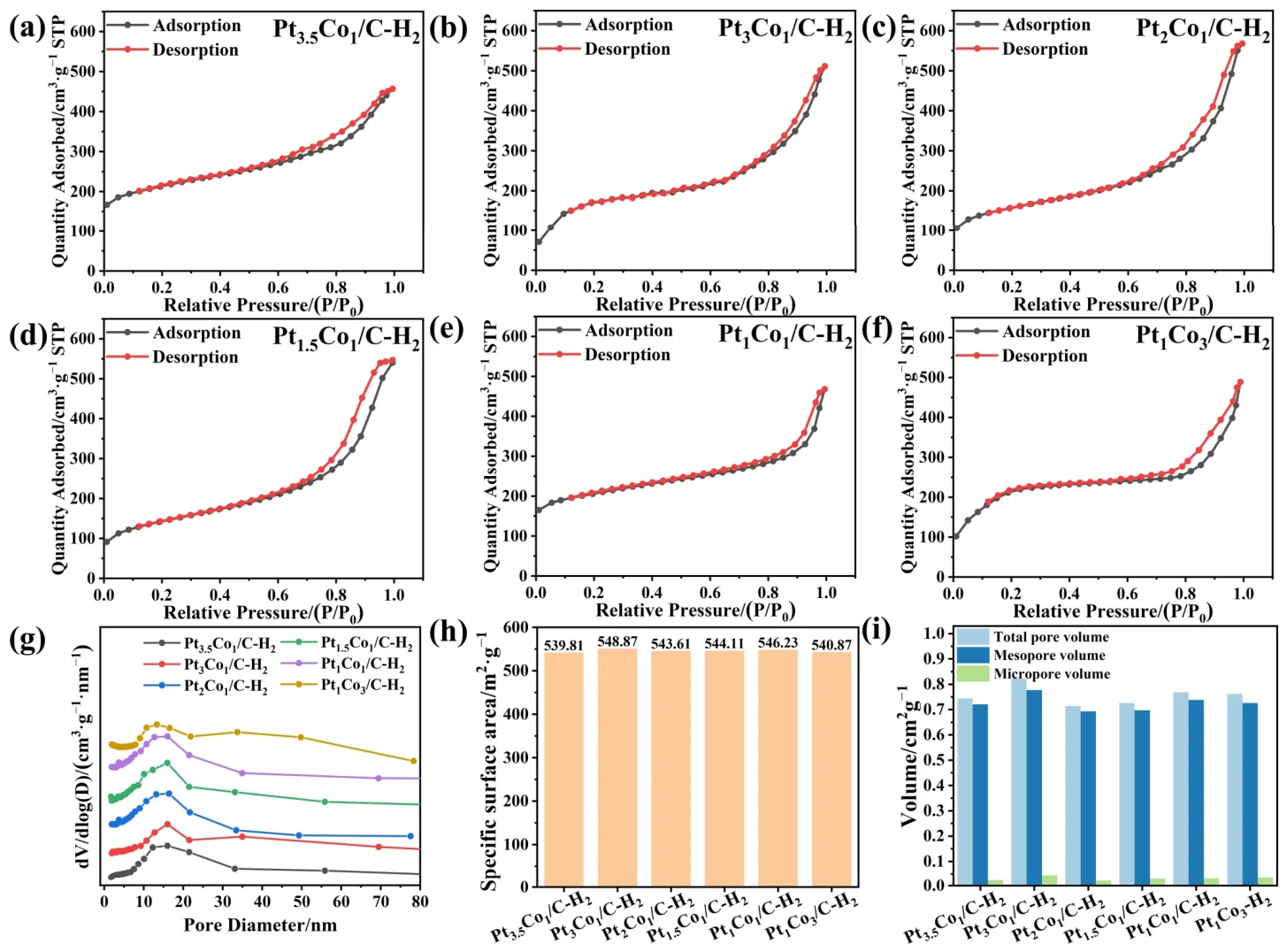
N_2_ adsorption–desorption isotherms and pore-structure analysis of Pt_x_Co_y_/C-H_2_ catalysts with varying Pt:Co ratios: (**a**–**f**) isotherms; (**g**) pore-size distributions; (**h**) BET surface areas; (**i**) micropore and mesopore volumes.

**Figure 3:**
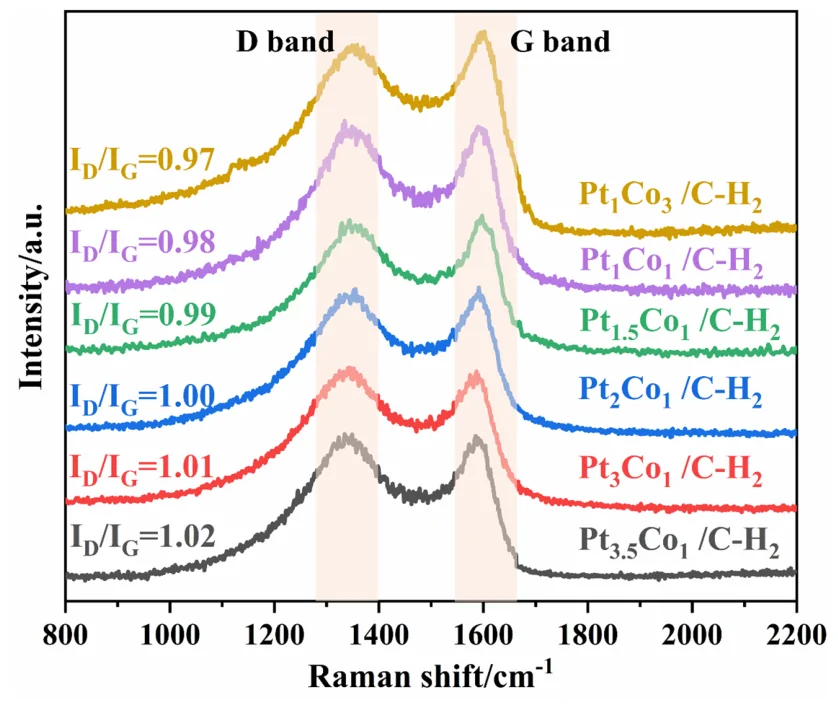
Raman spectra of Pt_x_Co_y_/C-H_2_ catalysts with varying Pt:Co ratios, showing characteristic D-band (1350 cm^−1^) and G-band (1590 cm^−1^) signals of the carbon support.

**Figure 4:**
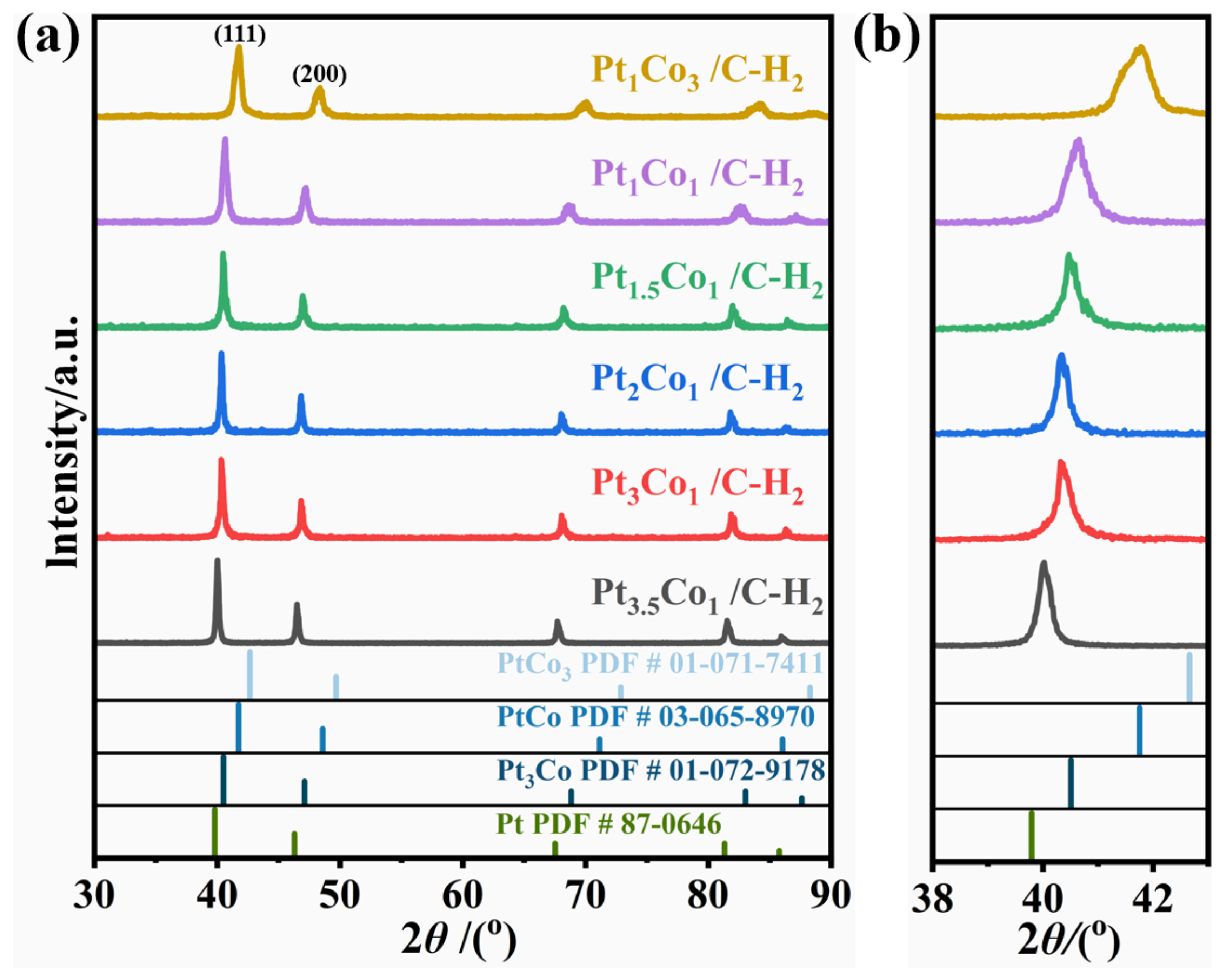
XRD patterns of Pt_x_Co_y_/C-H_2_ catalysts with varying Pt:Co ratios: (**a**) full-range XRD patterns; (**b**) magnified view of the (111) diffraction peak near 2θ = 40°, showing the peak shift associated with Pt-Co alloying.

**Figure 5:**
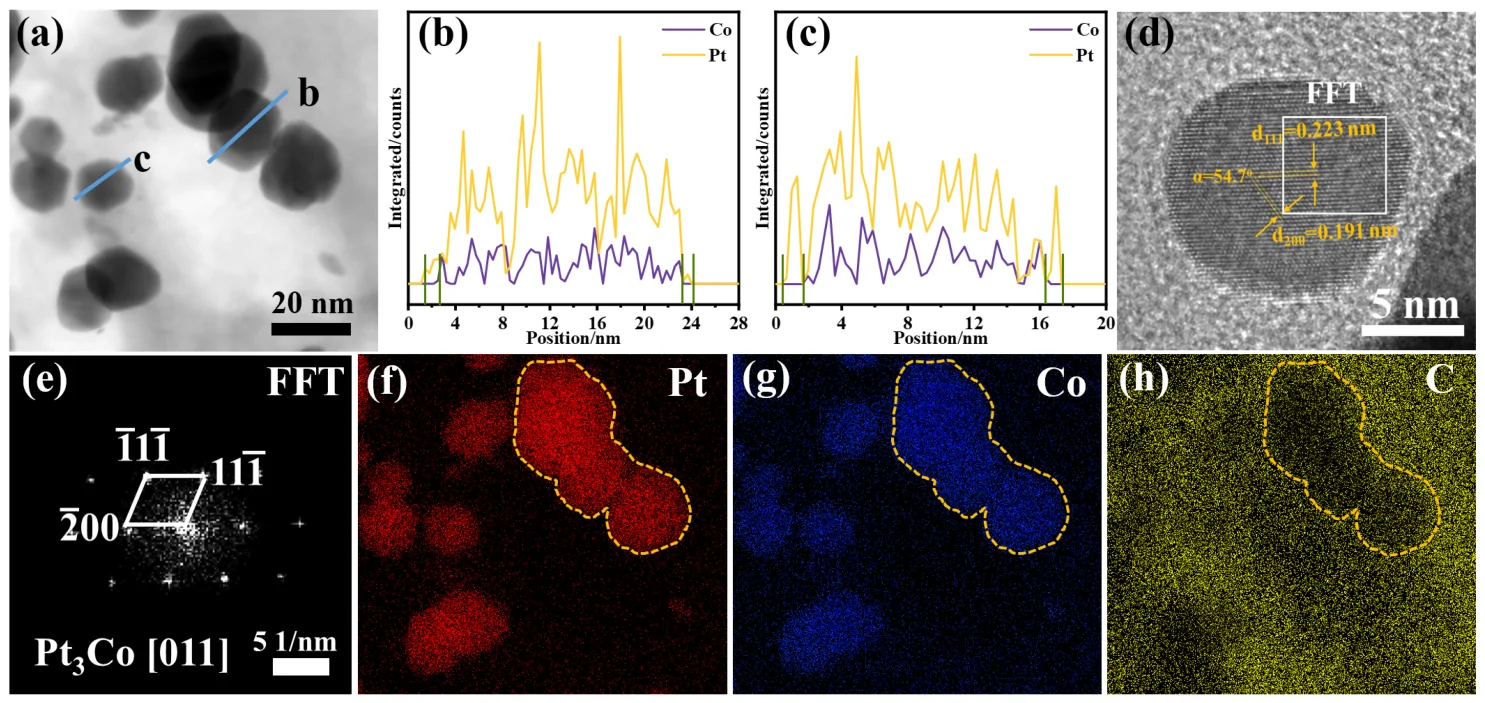
Morphology and structural characterization of Pt_3_Co_1_/C-H_2_ catalyst: (**a**) TEM image; (**b**,**c**) EDS line-scan profiles of Co and Pt; (**d**) HRTEM image with the region selected for FFT analysis indicated; (**e**) corresponding FFT pattern; (**f**–**h**) elemental mapping images of Pt, Co and C.

**Figure 6:**
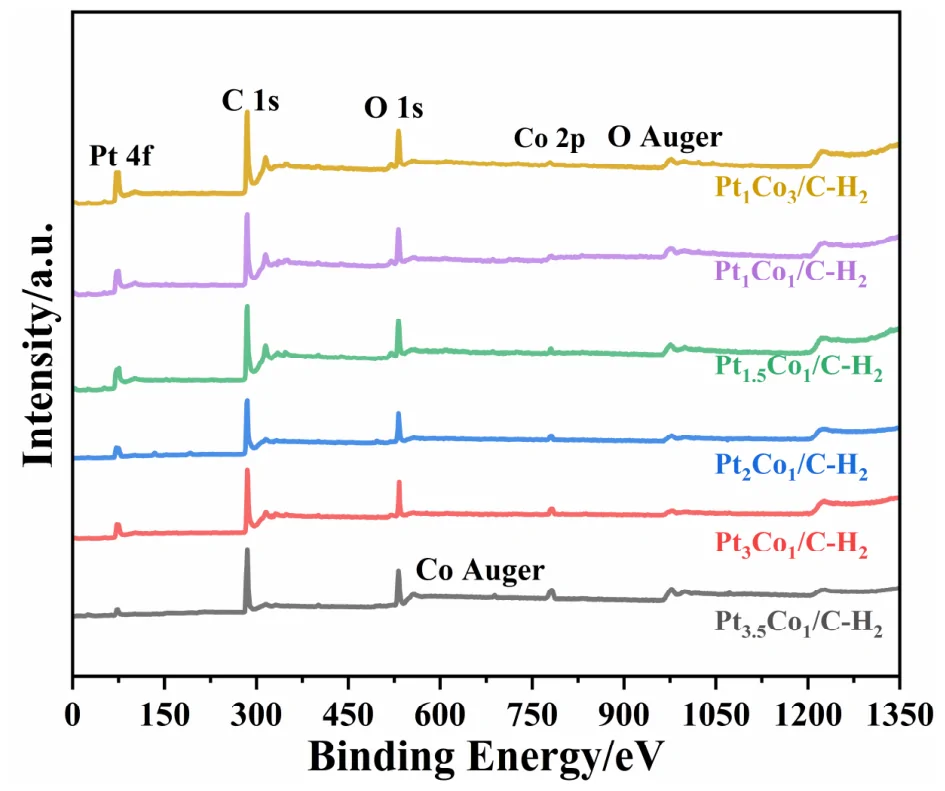
XPS survey spectra of Pt_x_Co_y_/C-H_2_ catalysts with varying Pt:Co ratios, showing signals of Pt, Co, C, and O.

**Figure 7:**
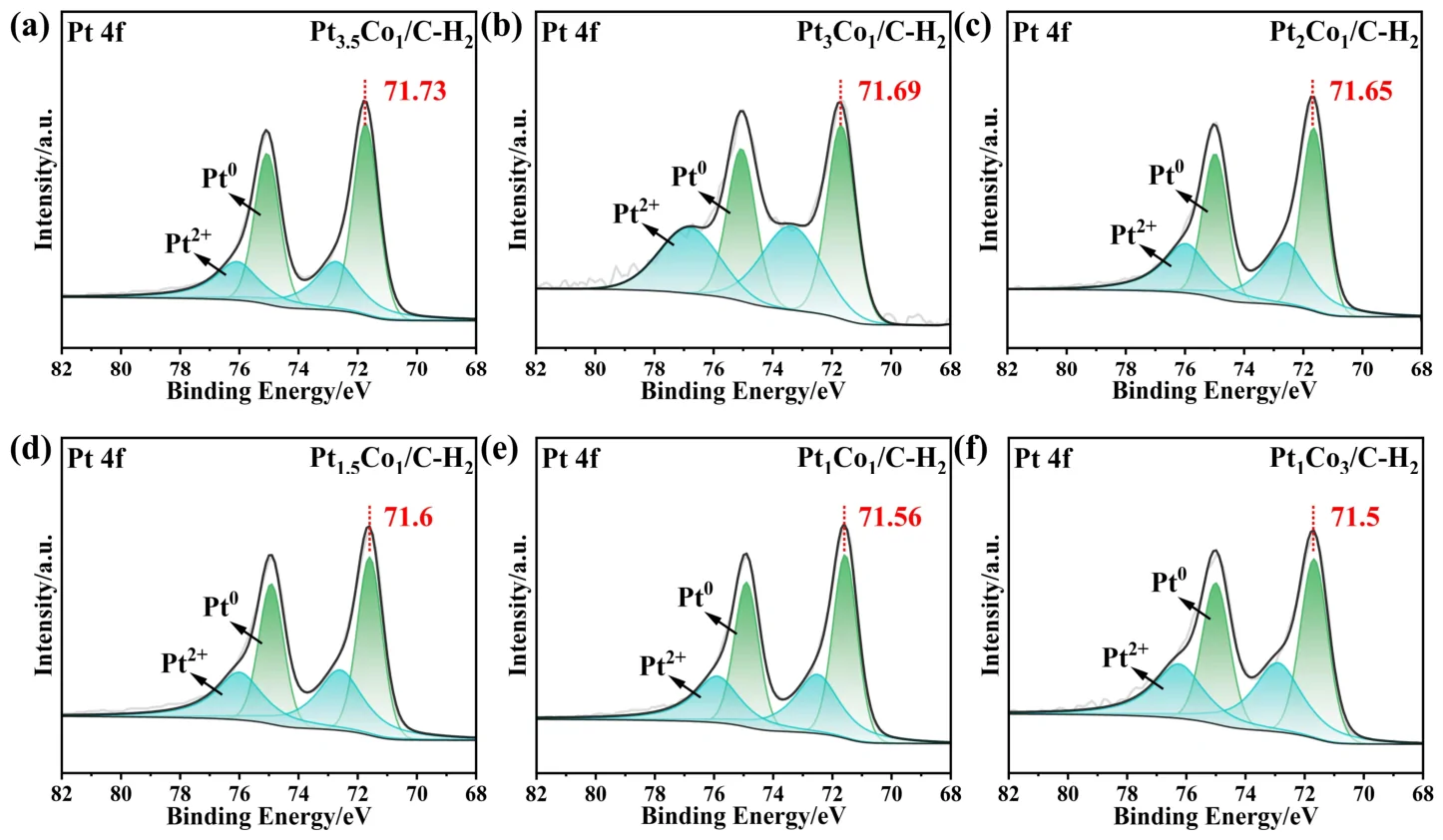
High-resolution Pt 4f XPS spectra of Pt_x_Co_y_/C-H_2_ catalysts with varying Pt:Co ratios, deconvoluted into Pt^0^ and Pt^2+^ components. (**a**) Pt_3.5_Co_1_/C-H_2_, (**b**) Pt_3_Co_1_/C-H_2_, (**c**) Pt_2_Co_1_/C-H_2_, (**d**) Pt_1.5_Co_1_/C-H_2_, (**e**) Pt_1_Co_1_/C-H_2_, and (**f**) Pt_1_Co_3_/C-H_2_.

**Figure 8:**
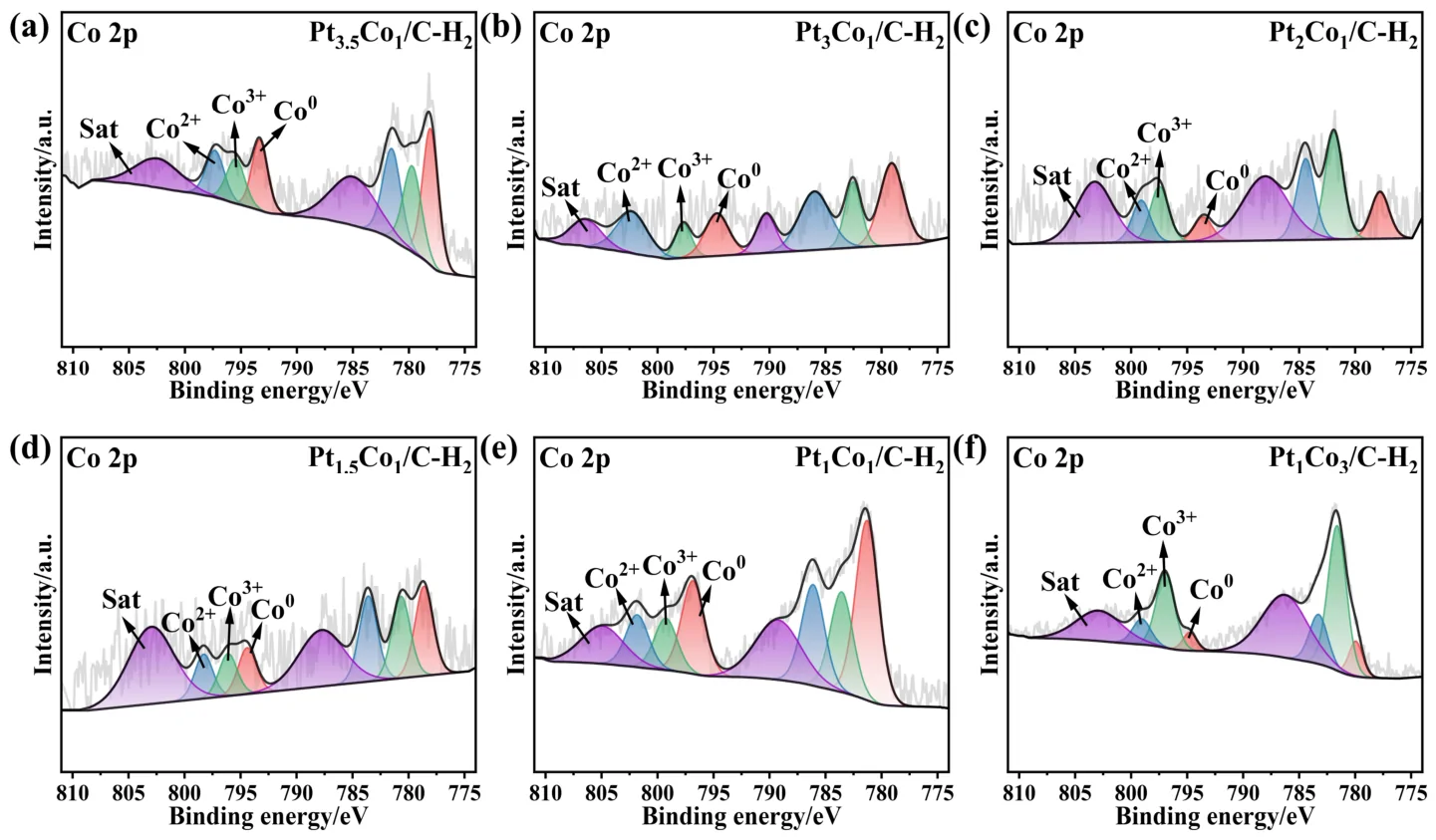
High-resolution Co 2p XPS spectra of Pt_x_Co_y_/C-H_2_ catalysts with varying Pt:Co ratios, deconvoluted into Co^0^, Co^2+^, Co^3+^, and satellite components. (**a**) Pt_3.5_Co_1_/C-H_2_, (**b**) Pt_3_Co_1_/C-H_2_, (**c**) Pt_2_Co_1_/C-H_2_, (**d**) Pt_1.5_Co_1_/C-H_2_, (**e**) Pt_1_Co_1_/C-H_2_, and (**f**) Pt_1_Co_3_/C-H_2_.

**Figure 9:**
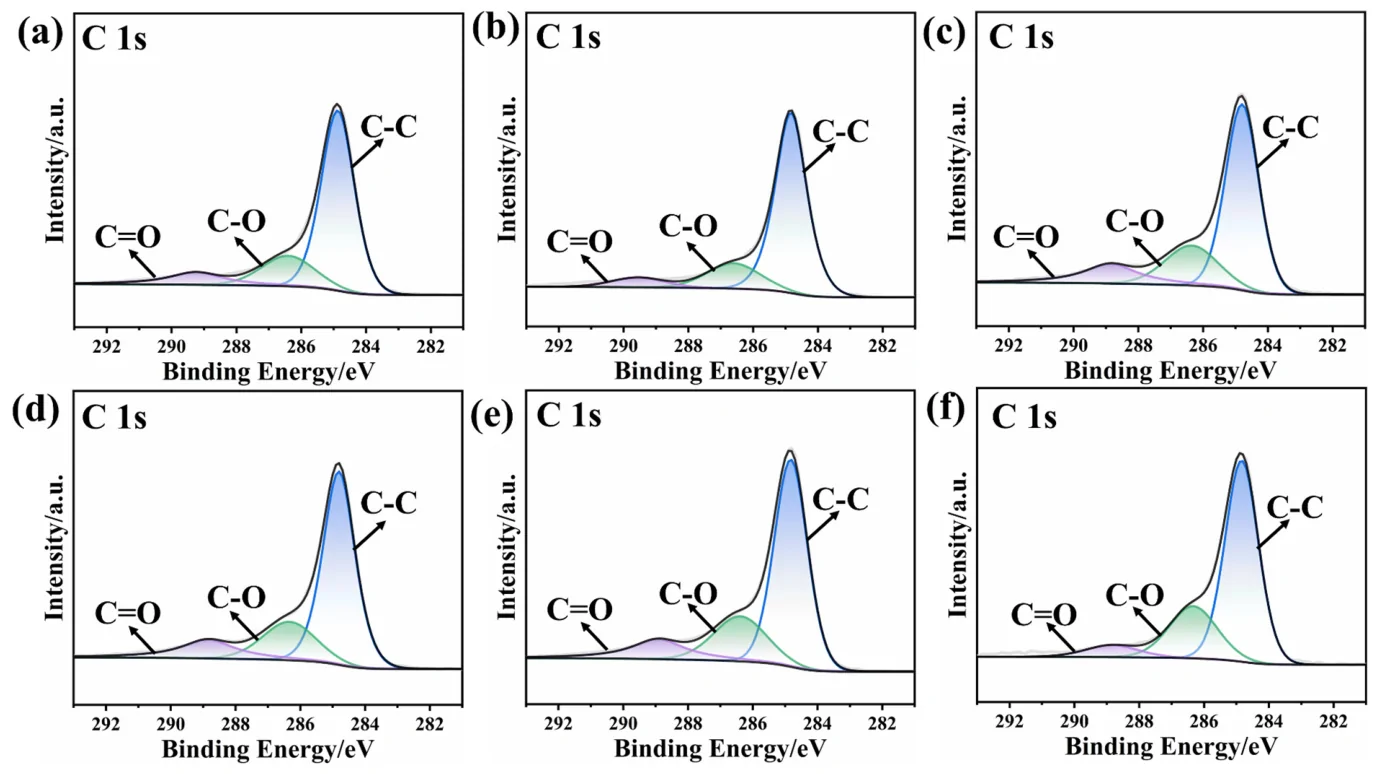
High-resolution C 1s XPS spectra of Pt_x_Co_y_/C-H_2_ catalysts with varying Pt:Co ratios. (**a**) Pt_3.5_Co_1_/C-H_2_, (**b**) Pt_3_Co_1_/C-H_2_, (**c**) Pt_2_Co_1_/C-H_2_, (**d**) Pt_1.5_Co_1_/C-H_2_, (**e**) Pt_1_Co_1_/C-H_2_, and (**f**) Pt_1_Co_3_/C-H_2_.

**Figure 10:**
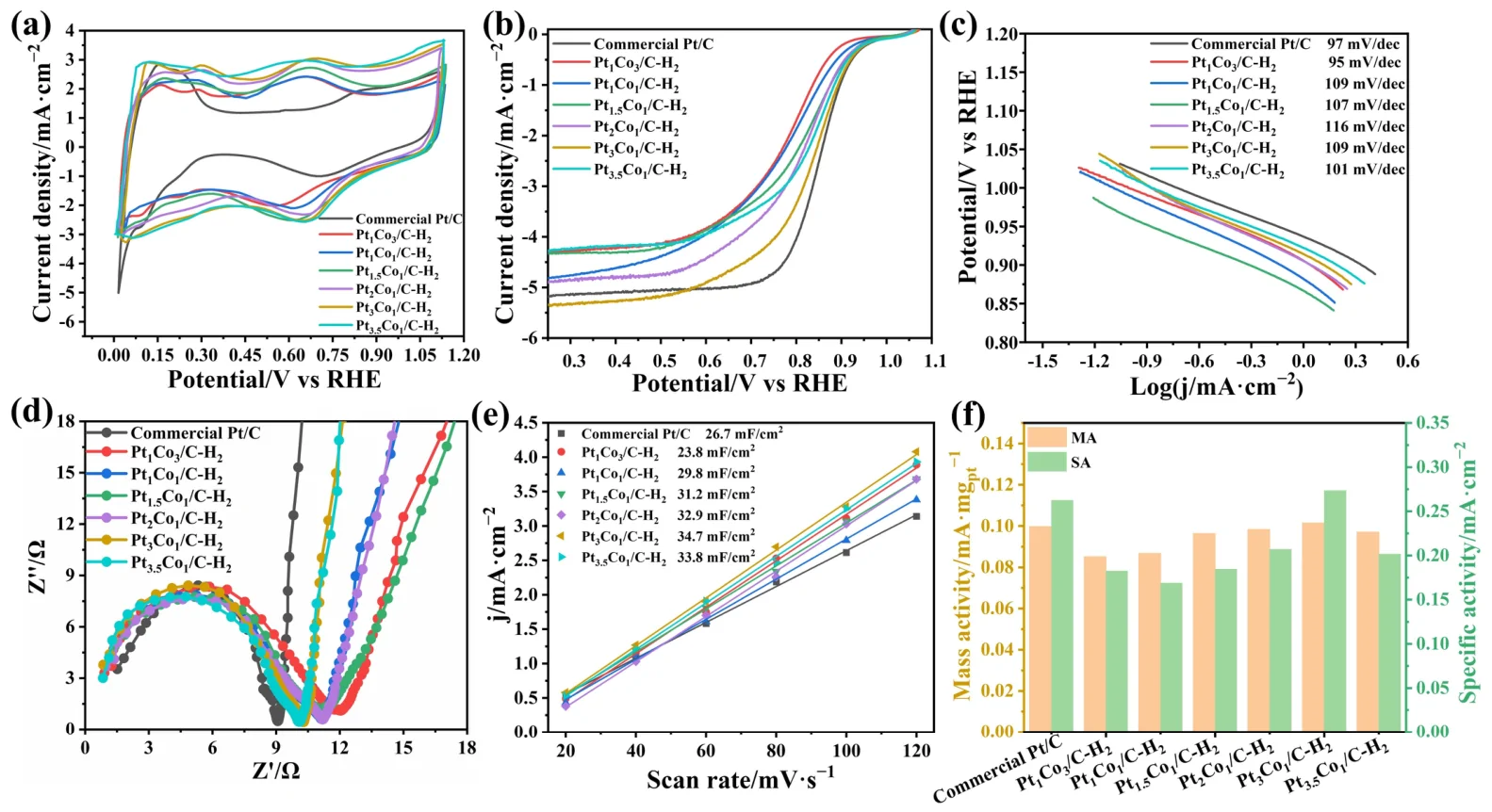
Electrochemical performance of Pt_x_Co_y_/C-H_2_ catalysts: (**a**) cyclic voltammograms (CVs) recorded in N_2_-saturated 0.1 M HClO_4_ at 50 mV·s^−1^; (**b**) linear sweep voltammograms (LSVs) recorded in O_2_-saturated 0.1 M HClO_4_ at 1600 rpm and 5 mV·s^−1^; (**c**) Tafel plots derived from the LSV data; (**d**) electrochemical impedance spectra (EIS) recorded at 0.85 V vs. RHE; (**e**) double-layer capacitance (C_dl_) as a function of scan rate; (**f**) mass activity (MA) and specific activity (SA) at 0.85 V vs. RHE.

**Figure 11:**
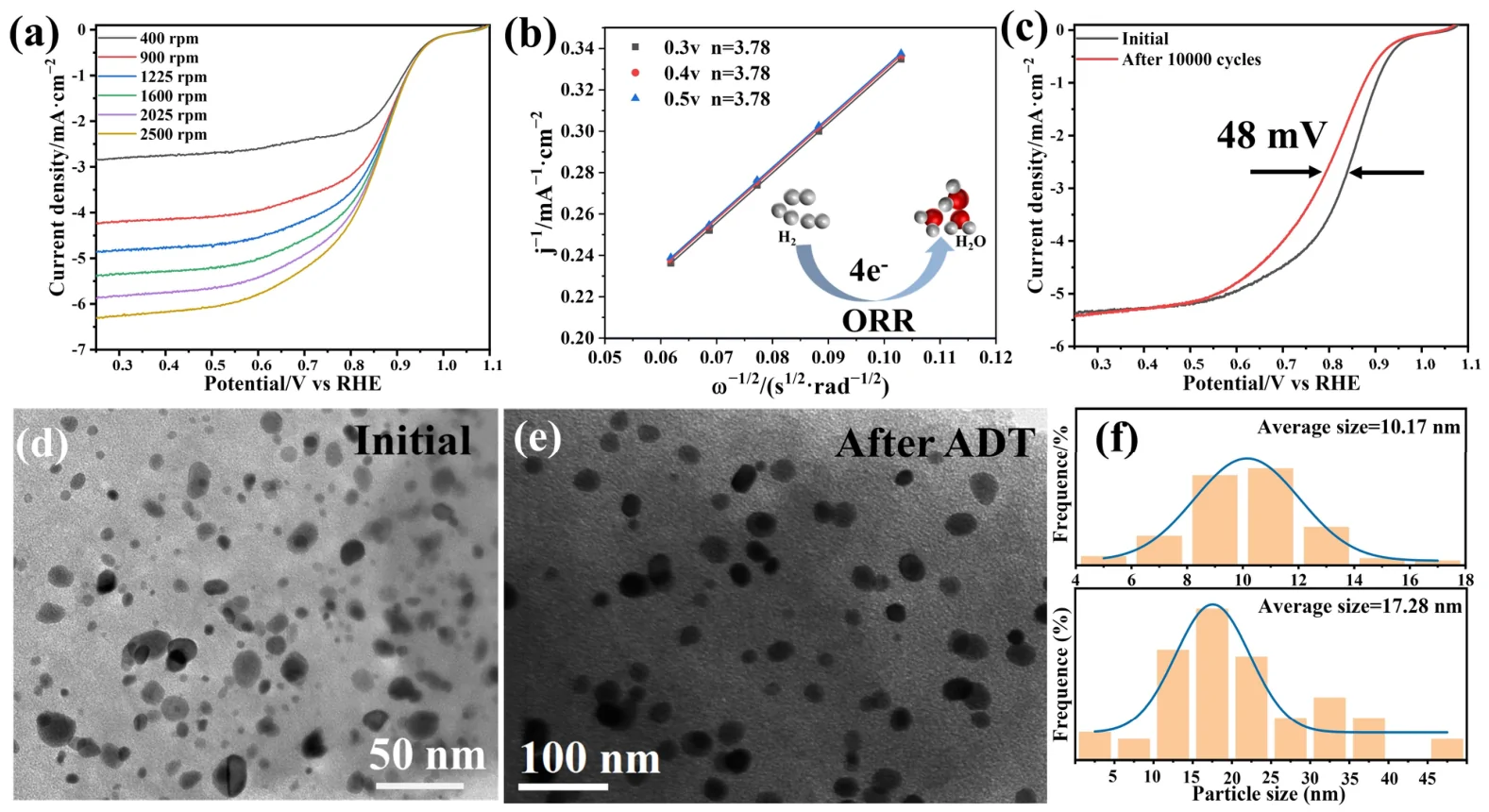
Electron-transfer mechanism and cycling stability of Pt_3_Co_1_/C-H_2_: (**a**) LSVs at various rotation rates (400–2500 rpm) in O_2_-saturated 0.1 M HClO_4_; (**b**) Koutecký–Levich (K-L) plots at 0.3–0.5 V vs. RHE; (**c**) LSVs before and after the ADT (10,000 cycles, 0.60–1.00 V); (**d**,**e**) TEM images before and after the ADT; (**f**) corresponding particle-size distributions.

## Data Availability

The original contributions presented in this study are included in the article/Supplementary Materials. Further inquiries can be directed to the corresponding author.

## Supplementary Materials

The following supporting information can be downloaded at: https://www.mdpi.com/article/10.3390/ma19214689/s1, Table S1: Surface elemental composition of Pt_x_Co_y_/C-H_2_ catalysts from XPS. Table S2: Surface elemental composition of Pt_x_Co_y_/C-Air catalysts from XPS.
