# Supplementary material for: H2-Annealed Core–Shell Pt-Co/C Electrocatalysts with Enhanced Durability for the Oxygen Reduction Reaction
Source: Materials (Basel). 2026 Nov 4;19(21):4689. doi: 10.3390/ma19214689 (PMC13765265; doi:10.3390/ma19214689)
Supplement: Supplementary file 1 [file materials-19-04689-s001.zip › materials-4637195-supplementary.pdf]

Supplementary Information to:

H_2-Annealed Core-Shell Pt-Co/C Electrocatalysts with Enhanced Durability for the Oxygen Reduction Reaction

Bingguo Xue [1], Wei Wang [1], Yuxiang Dai [1], Shaohong Liu [1,2,*], Limin Zhou [2], Hao Cui [2], Hongying Pei [3], Wenyan Zhou [3], Manmen Liu [3], Haigang Dong [2], Ming Wen [2], Li Chen [2] and Song Li [1]

[1] Key Laboratory for Anisotropy and Texture of Materials (Ministry of Education), School of Materials Science and Engineering, Northeastern University, Shenyang 110819, China
[2] State Key Laboratory of Precious Metal Functional Materials, Yunnan Precious Metals Laboratory Co., Ltd., Kunming 650106, China
[3] Sino-Platinum Metals Semiconductor Materials (Yunnan) Co., Ltd., Kunming 650106, China
* Correspondence: liush@smm.neu.edu.cn

Table S1. Surface elemental composition of $Pt_xCo_y/C-H_2$ catalysts from XPS.

	Pt/at.%	Co/at.%	Pt:Co	C/at.%	O/at.%	O/C
$Pt_1Co_3/C-H_2$	1.22	0.47	2.60:1	85.05	13.22	0.16
$Pt_1Co_1/C-H_2$	4.67	1.00	4.67:1	85.72	9.59	0.11
$Pt_{1.5}Co_1/C-H_2$	1.46	0.32	4.56:1	83.53	14.69	0.18
$Pt_2Co_1/C-H_2$	5.36	0.85	6.31:1	78.66	15.13	0.19
$Pt_3Co_1/C-H_2$	4.87	1.50	3.25:1	78.22	15.41	0.20
$Pt_{3.5}Co_1/C-H_2$	5.07	0.75	6.76:1	79.53	14.65	0.18

Table S2. Surface elemental composition of Pt_xCo_y/C-Air catalysts from XPS.

	Pt/at.%	Co/at.%	Pt:Co	C/at.%	O/at.%	O/C
Pt_1Co_3/C-Air	0.21	0.74	0.28:1	51.23	47.82	0.93
Pt_1Co_1/C-Air	0.63	0.54	1.17:1	52.03	46.80	0.90
$Pt_{1.5}Co_1/C$-Air	0.67	0.45	1.49:1	53.96	44.92	0.83
Pt_2Co_1/C-Air	0.72	0.38	1.89:1	52.26	46.66	0.89
Pt_3Co_1/C-Air	0.91	0.34	2.68:1	52.06	46.69	0.90
$Pt_{3.5}Co_1/C$-Air	1.12	0.27	4.15:1	56.05	42.56	0.76
